# A single-center, single-blinded, randomized, parallel-group, non-inferiority trial to compare the efficacy of a 22-gauge needle versus a 15 blade to perform an Achilles tendon tenotomy in 244 clubfeet—study protocol

**DOI:** 10.1186/s13063-023-07728-9

**Published:** 2023-10-31

**Authors:** Manon Pigeolet, Jabbar Ghufran Syed, Sadia Ahmed, Muhammad Amin Chinoy, Mansoor Ali Khan

**Affiliations:** 1 Faculty of Medicine, Université Libre de Bruxelles, Brussels, Belgium; 2 Department of Pediatric Orthopedics, Hôpital Necker – Enfants Malades, Paris Cité University, Paris, France; 3 Department of Orthopedic Surgery, The Indus Hospital, Korangi Campus, Karachi, Pakistan

**Keywords:** Clubfoot, Global surgery, Pediatrics, Orthopedics, Ponseti treatment

## Abstract

**Background:**

Achilles tendon tenotomy is an integral part of the Ponseti method, aimed at correcting residual equinus and lack of dorsiflexion after correction of the adductus deformity in clubfoot. Percutaneous tenotomy using a number 15 scalpel blade is considered the gold standard, resulting in excellent results with minimal complications. The use of a large-bore needle to perform Achilles tendon tenotomies has been described in literature, but a large-scale randomized controlled trial is currently lacking. In this trial, we aim to show the non-inferiority of the needle tenotomy technique compared to the gold standard blade tenotomy technique.

**Methods:**

We will randomize 244 feet into group A: needle tenotomy or group B: blade tenotomy. Randomization will be done using a block randomization with random block sizes and applying a 1:1 allocation to achieve an intervention and control group of the exact same size. Children will be evaluated at 3 weeks and 3 months post-tenotomy for primary and secondary clinical outcomes. The primary clinical outcome will be the range of dorsiflexion obtained the secondary clinical outcomes will be frequency of minor and major complications and Pirani score. The non-inferiority margin was set at 4°, and thus, the null hypothesis of inferiority of the needle technique will be rejected if the mean difference between both techniques is less than 4°. The statistical analysis will use a multi-level mixed effects linear regression model for the primary outcomes and a multi-level mixed effects logistic regression model for the secondary clinical outcomes. The physician performing the evaluations post-tenotomy will be the only one blinded to group allocation.

**Trial registration:**

This trial was registered prospectively with ClinicalTrials.gov registration number: NCT04897100 on 21 May 2021.

**Supplementary Information:**

The online version contains supplementary material available at 10.1186/s13063-023-07728-9.

## Introduction

### Background and rationale

#### Background

Achilles tendon tenotomy is an integral part of the Ponseti method, aimed at correcting residual equinus and lack of dorsiflexion after correction of the adductus deformity in clubfoot [1]. Achilles tendon tenotomy is required in 70–80% of all patients after a full cycle of castings has been completed [1, 2]. Percutaneous tenotomy using a number 15 scalpel blade is the gold standard, which can be performed in an outpatient setting under local anesthesia [1, 3]. A complication rate of 2% (predominantly neurovascular injuries) has been reported in literature, with accidental sectioning of the peroneal artery being the most common complication [4]. Development of a pseudo-aneurysm after accidental sectioning of the peroneal artery has been reported in a case report; which delayed further clubfoot treatment [5]. Blade tenotomy is a highly successful technique to correct insufficient dorsiflexion in clubfoot patients with about 4% of patients who do not reach the required 15° dorsiflexion, 1 year after the tenotomy requiring a re-tenotomy [1, 6, 7].

Severity of the clubfoot deformity and clinical progress in treatment of the clubfoot using the Ponseti technique can be evaluated with the Pirani score. The Pirani score is a clinical score consisting of a clinical evaluation of midfoot contracture and hindfoot contracture severity. Total score lays between 0 and 6 with 6 representing a severely malformed foot and 0 a normally appearing foot [8].

Percutaneous needle tenotomy is an established technique in orthopedics and has been described as a safe intervention for multiple purposes, including Achilles tendon tenotomy [9–12]. It is used for quadriceps tendon tenotomy in congentinal knee dislocation in newborns [9] and for superficial tendon tenotmies, in stroke patients with muscle contractures [10] among others. No contraindications have been reported for this technique in the literature [9–11].

Percutaneous needle tenotomy has been widely described in literature as an alternative technique to the blade tenotomy with very favorable results in a population before walking age [11–15]. The original technique described by Minkowitz et al. uses a large-gauge (16–19 G) needle to percutaneously cut the Achilles tendon [12, 15]. We adapted the technique for use in a low-resource setting using a 22-gauge needle [16]. Although bleeding has been reported following this technique with similar rates as for the percutaneous blade technique, no major complications have been reported in the literature as yet [12, 15]. The needle tenotomy technique is easy to use and easy to teach to clinicians who are new to the Ponseti treatment and shown to instill less anxiety in parents and caregivers compared to a blade tenotomy [15].

Inaccessibility of Ponseti clubfoot care and non-adherence to the Ponseti protocol remain big challenges in low- and middle-income countries [17, 18]. Lack of providers, long distance to clinics, and parental fear for the tenotomy are major underlying issues generating lack of access to Ponseti care [15, 17, 18]. Introducing needle tenotomies in areas with a low density of orthopedic providers by training non-surgeons to perform the tenotomy may be a gamechanger in making Ponseti care more accessible throughout the world.

The majority of reports on the needle tenotomy technique constitute case series without an active comparator [11]. To date, only one small randomized control trial comparing percutaneous needle versus blade tenotomy has been published including 55 feet [13]. The authors did not find any significant statistical difference between the two groups (*p* > 0.05). No information detailing achieved dorsiflexion post-tenotomy, the demographics of the included participants, how the sample size was determined, nor the randomization strategy were reported. A correctly sampled non-inferiority trial comparing needle tenotomy and blade tenotomy is necessary to establish the non-inferiority of the needle tenotomy to assure that introducing this technique around the globe is safe, ethical, and effective.

#### Rationale

The Ponseti method has become the gold standard treatment for clubfoot globally. It involves an Achilles tendon tenotomy in most patients, which is usually done with a blade in the majority of settings. In low-resource settings, a needle tenotomy has shown to be a technique easy to teach, with little complications and a good acceptance rate among parents and caregivers. By showing the non-inferiority of the needle technique compared to the blade technique in terms of complications and dorsiflexion range post-tenotomy, we hope to increase the technique’s usage among Ponseti providers, especially so in settings where the Ponseti treatment is provided by non-orthopedic surgeons or non-physicians, and increase access to clubfoot care in low-resource settings.

### Objectives and hypotheses

#### Objectives

Through this study, we aim to compare the clinical outcomes in children receiving either a blade or a needle percutaneous tenotomy.

*Main research questions*: Is there a difference in clinical outcome (dorsiflexion range) after an Achilles tendon tenotomy performed with a blade or needle at 3 months post-operatively?

*Secondary research questions*: Is there a difference in secondary clinical outcomes?

Secondary clinical outcomes include the following: minor and major complication frequency and types and Pirani score at 3 months post-operatively.

#### Hypotheses

*Main hypothesis*: Percutaneous needle tenotomy has a non-inferior clinical outcome compared to the use of a percutaneous blade tenotomy when comparing the patient’s dorsiflexion range.

*Secondary hypothesis*: Percutaneous needle tenotomy complication frequency and Pirani score are clinically comparable in frequency of complication (minor and major) and Pirani score to the percutaneous blade tenotomy.

## Methods

### Study setting

In order to establish non-inferiority of the needle tenotomy technique, a sufficient large enough sample size needs to be obtained. Additionally, up to 90% of children with clubfoot are born in a low- or middle-income country (LMIC), making an LMIC the culturally and environmentally most appropriate setting to generalize results beyond our center. Given the high volume of children with clubfoot who receive treatment at the Pehla Qadam clubfoot clinic at the Indus Hospital & Health Network, this center was selected for the implementation of this trial. The trial is being conducted at Pehla Qadam clubfoot clinic, The Indus Hospital, Korangi campus, Karachi, Pakistan.

### Trial design

This trial is designed as a randomized, controlled, single-blinded, single-center non-inferiority trial with two parallel groups and a primary endpoint of achieved dorsiflexion at 3 months after tenotomy. Randomization will be performed as block randomization with a 1:1 allocation.

### Patient timeline

**Fig. 1:**
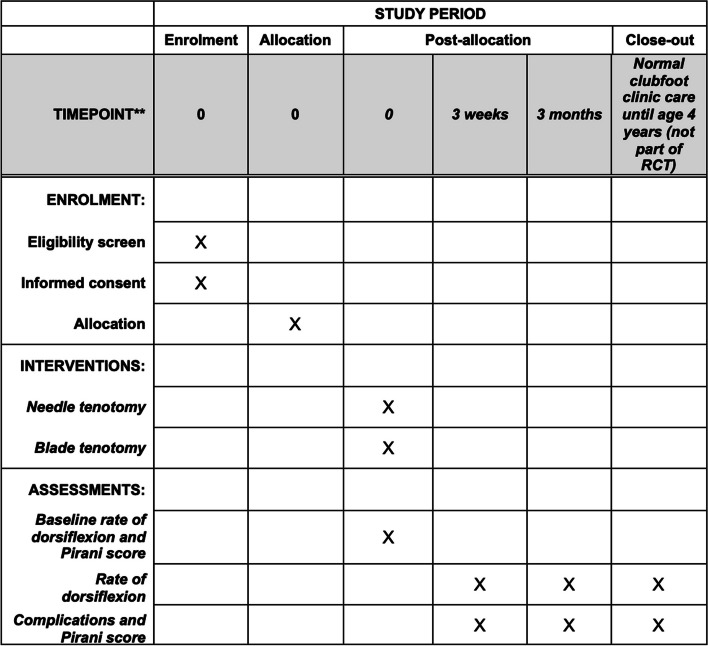
SPIRIT figure trial overview

**Fig. 2:**
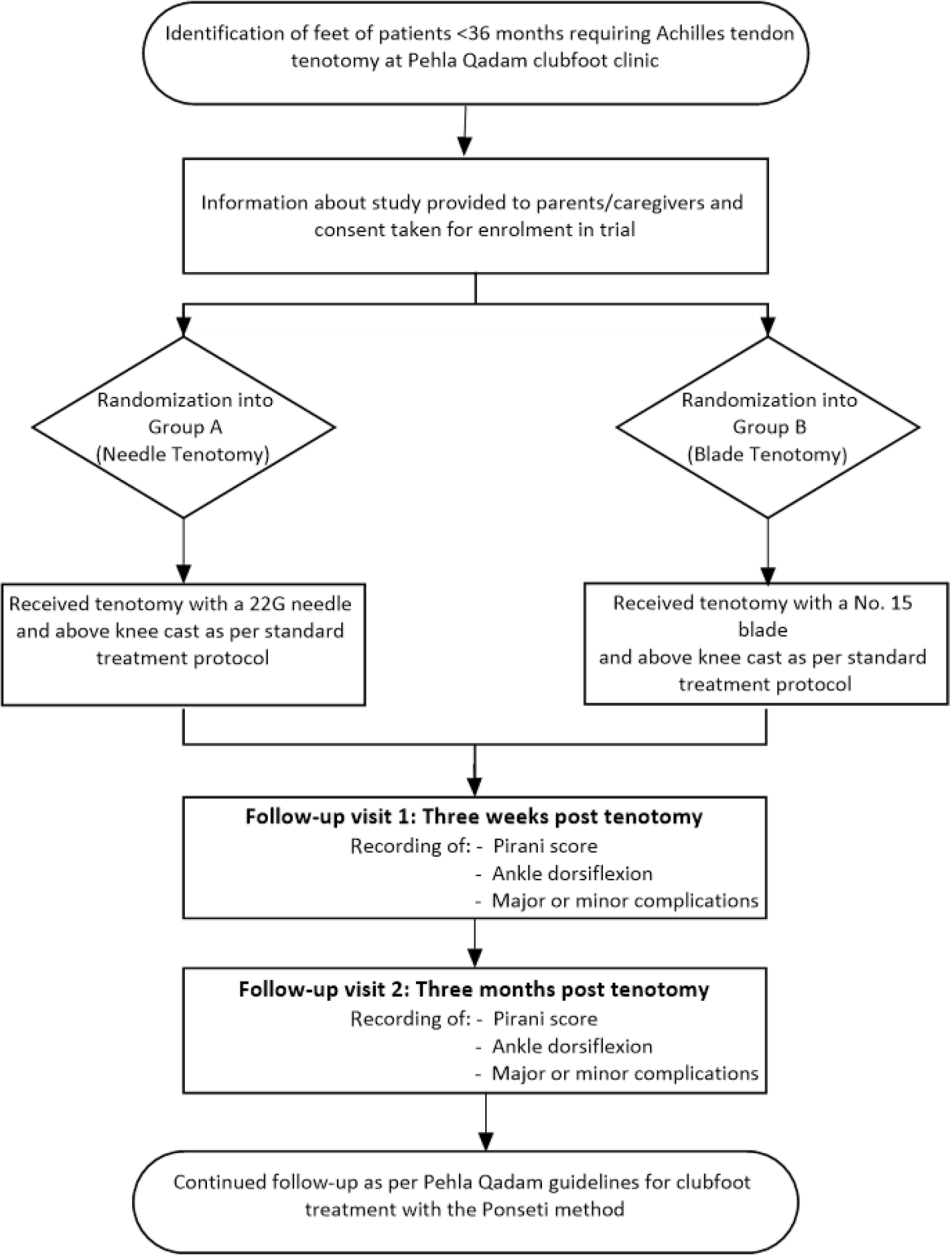
Trial process flow chart

Patients enrolled in this trial are not required to attend any additional visits beyond the visits required as part of the standard treatment protocol at Pehla Qadam clubfoot clinic. Randomization will happen at the moment the indication for an Achilles tendon tenotomy is set. The patient timeline is visualized in Fig. 1 using a SPIRIT chart and in Fig. 2 using a process flow chart.

### Consent or assent

#### Obtaining informed consent

The program coordinator for the Pehla Qadam clubfoot clinic is also the program coordinator for this trial. She evaluates patients’ eligibility into the Pehla Qadam clubfoot program during their first appointment at the clinic. If they are eligible to be enrolled as patients, the program coordinator will inform them about the ongoing studies and trials at the Pehla Qadam clubfoot clinic and inform them for which studies they are currently eligible or could potentially become eligible in the future.

If the clinical need for an Achilles tendon tenotomy is established by the treating physician, the program coordinator is informed of this potential candidate for the trial. The child’s parents/caregiver will be invited by the program coordinator to discuss the trial and potential enrollment. After eligibility for the trial is confirmed and an informed discussion has taken place, the program coordinator will obtain informed consent from the parents/caregiver and the child will receive its tenotomy the same day. The information sheet and the informed consent form will be read out loud by the program coordinator during the informed consent taking to assure that parents/caregivers have understood everything in the document before signing it. A copy of the information sheet and informed consent form are available in English and in Urdu and will be provided to the parents/caregiver. The English version of the information sheet and informed consent form is available in Additional file 1.

### Eligibility criteria

All patients under the age of 36 months where the treating physician has established the clinical need for an Achilles tendon tenotomy will be considered for inclusion. The decision to propose an Achilles tendon tenotomy to a patient has to happen in the best interest of the patient and can never be influenced by a demand of the study investigator.

Inclusion criteria

- Parental consent to enroll child in the study
- Idiopathic clubfoot
- Age less than or equal to 36 months at the time of tenotomy
- Enrolled at the Pehla Qadam clinic at The Indus Hospital in Karachi
- Fully corrected adductus and equinus deformity after serial casting
- Insufficient degree of dorsiflexion (< 15°)

Exclusion criteria

- Refusal of parents to enroll child into this study
- Syndromic clubfoot
- Previous treatment for clubfoot (surgical or non-surgical) received
- Underlying medical conditions unrelated to clubfoot that may serve as a contra-indication; this decision will be left on the discretion of the treating orthopedic surgeon

### Sample size

#### Change of sample size after initial protocol creation

The sample size for this trial has been adapted after submission of the initial protocol. In preparation of submission of the final protocol for publication, it was brought to our attention that a sample size calculation for a non-inferiority trial requires the determination of a non-inferiority margin. The initial sample size, which had been calculated based on an expected difference in success rate between both tenotomy techniques, has been abandoned and replaced by a sample size calculation based on the calculation of an inferiority margin. The full calculation can be found below.

#### Non-inferiority margin determination

Dorsiflexion was defined as “true dorsiflexion” at the ankle joint and not mobility at the midfoot level. True maximal dorsiflexion measures the tibiocalcaneal angle when the foot is put in maximum dorsiflexion. Only a limited number of studies report on the range of dorsiflexion achieved after tenotomy. We identified two studies that used post-tenotomy radiographs to assess for dorsiflexion range [19, 20]. One study assessed evolution of ankle dorsiflexion over time post-tenotomy. The study states that children who still maintained 15° dorsiflexion at age 12 all had a minimum of 20° dorsiflexion under the age of 3 years [21]. This raises the question if the ideal dorsiflexion under the age of 3 years should be 20° instead of 15° as initially proposed by Ponseti. We identified 17.5° dorsiflexion, mid-way between both proposed cut-offs, minimally acceptable range of post-operative dorsiflexion in our study.

**Fig. 3:**
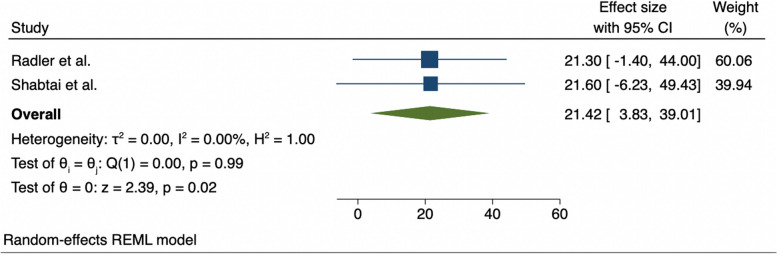
Inverse-variance model with random effects to determine mean dorsiflexion rate using blade tenotomy

The mean range of dorsiflexion and standard deviation including the data of both radiologic studies mentioned above was determined using an inverse-variance model with random effects (Fig. 3). The obtained mean range was 21.42° of dorsiflexion. The mean range of dorsiflexion obtained from the literature is very close to the mean range of dorsiflexion (20.2°) obtained in our pilot study before starting the full RCT.

$$\begin{array}{c}\mathrm{Non}-\mathrm{inferiority \,margin}\\ =\mathrm{ mean \,range \,of\, dorsiflexion \,from\, literature }-\mathrm{ minimally\, acceptable\, range\, of\, post}-\mathrm{operative\, dorsiflexion}\\ = 21.42^\circ - 17.5^\circ \\ = 3.92^\circ \end{array}$$

For practical purposes, a non-inferiority margin of 4° will be applied instead of 3.92.

#### Sample size and power calculation

Sample sizes are calculated using the WHO sample size determination software [22].

Sample size calculation for primary outcome:

- Alpha = 5%
- Power 95%
- Non-inferiority margin: 4
- Standard deviation: 9.02

Required sample size = 222 feet (111 in each arm).

Drop-out from clubfoot programs is a common event in LMICs, especially throughout the bracing phase. In literature, drop-out rates of up to 30% have been reported over the total period of bracing which is about 3 years on average [17, 18, 23]. Internal data from the Pehla Qadam clubfoot program shows similar outcome with about 30% drop-out over the entire course of the treatment. We therefore expect during the first year of follow-up to lose about 10% of our patients. We will therefore increase the sample size by 10% to adjust for the expected drop-out.

A total sample size of 244 feet (122 per arm) will be recruited for this trial.

### Outcomes

Patients will be followed as per the standard follow-up schedule at the Pehla Qadam clubfoot clinic. Patients will be evaluated at 3 weeks and at 3 months post-tenotomy during regular follow-up consultations. The dorsiflexion range at 3 months post-tenotomy is considered the primary outcome. These measurement points were chosen as they coincide with the standard follow-up appointments established at the Pehla Qadam clubfoot clinic. Given that long-term follow-up is challenging in low-resource settings, the primary outcome measure was the first follow-up visit after cast removal, i.e., 3 months post-tenotomy to minimize drop-out from the trial.

The primary outcome measure is as follows: range of dorsiflexion will be registered at both follow-up visits.

The secondary outcome measures are as follows: complications including neurovascular damage in the heel region, skin, and wound problems or infections will be recorded at both follow-up visits. Neurovascular damage, an infection requiring antibiotic treatment or any other type of complication that requires the consultation of another medical specialty, will be considered a major complication. Scar hypertrophy, local edema, or erythema will be considered minor complications.

### Interventions

#### Interventions per study arm

Eligible feet will be randomized 1:1 between group A: 22-gauge needle and group B: 15-blade group for their Achilles tendon tenotomy. Group A will undergo an Achilles tendon tenotomy with a 22-gauge needle according to our previously described protocol. The complete surgical technique can be found in Syed et al. [16] Group B will have an Achilles tendon tenotomy according to the standard protocol using a blade. A complete description and overview of the standard technique using a blade can be found in Mosca et al. [24].

Both the needle tenotomy and the blade tenotomy are performed with the patient awake and in supine position. Local anesthesia (lidocaine 1%) is injected 1 cm above the insertion of the Achilles tendon on the calcaneum, 5 min before the tenotomy is performed. The heel is disinfected with betadine, and the foot is kept maximally dorsiflexed so that the Achilles tendon is taut.

If the patient’s foot is randomized to group A, the provider will introduce a 22-gauge needle perpendicular to the coronal plane 1 cm above the insertion of the Achilles tendon on the calcaneum. The tendon fibers will be cut using the tip of the needle in a medial to lateral fashion. After about 2–3 “cuts,” all tendon fibers will be cut, and the provider will appreciate the typical “pop” when the tendon is cut completely.

Damage to the neurovascular bundle is avoided by mimicking the blade tenotomy technique with the needle, which has proven to be safe. By introducing the needle from the medial side of the ankle at the level of the Achilles tendon, the needle is introduced at about 1 cm distance of the medial neurovascular bundle. This way the chance of the needle damaging the neurovascular bundle is much lower because the needle is being introduced in a direction away from the neurovascular bundle. Secondly, the cutting movement is made from in anterior to posterior fashion only when the needle tip is clearly touching the tendon. This way, the cutting movements are away from the neurovascular bundle and any brisk or uncontrolled movement would result in the needle perforating the lateral skin or the posterior skin of the heel but not towards the neurovascular bundle.

If the patient’s foot is randomized to group B, the provider will perform the standard blade tenotomy as described by Ponseti [1]. The blade is introduced into the tendon in a perpendicular way from the medial side. The blade is held parallel to the coronal plane during insertion and rotated 90° once inserted into the tendon. The tendon is then cut with one controlled cut of the blade from cutting from the deep to the superficial part of the tendon. The provider will appreciate the typical “pop” when the tendon is cut completely.

After the tenotomy, irrespective of the technique, a sterile gauze will be put over the insertion site where the tenotomy was done. The patient then receives the standard above-knee cast with the foot put in maximal dorsiflexion. The cast is removed after 3 weeks according to standard protocol. At 3 weeks and 3 months post-tenotomy, ankle dorsiflexion and Pirani scoring are measured as per standard protocol at the Pehla Qadam clubfoot clinic.

#### Choice of comparators

The comparator chosen in this study to proof non-inferiority of the needle tenotomy technique is the gold standard 15-blade technique. We chose to compare with the gold standard to assure that participants in this trial receive appropriate care for their clubfoot and to avoid over-estimating the efficacy of the needle tenotomy. If non-inferiority of the needle tenotomy compared to the gold standard can be proven, we can assure that patients who would receive a needle tenotomy did not receive an inferior or mediocre care compared to the care that they could be receiving if they were to receive a tenotomy with a 15 blade.

We opted for a use of a 22-gauge needle, as described in our surgical technique adapted to low-resource settings [16]. A 22-gauge needle is readily available in the clubfoot clinic, of low cost, and will leave a smaller scar mark while still offering sufficient rigidity to safely cut the tendon.

#### Modification in allocated interventions

Cross-over between groups is not allowed in the trial. If a change of intervention is deemed necessary by the treating physician after group allocation, the patient will be removed from the trial and receive the appropriate care deemed necessary by their treating physician.

#### Adherence to treatment in trial participants

Adherence to casting and brace wearing after tenotomy is a paramount to the overall success of the Ponseti treatment [1]. All patients at the Pehla Qadam clubfoot clinic receive counseling about the importance of adherence to the prescribed treatment. Patients in the trial will not receive any additional interventions to increase adherence beyond what is already offered as part of the standard treatment regimen.

#### Concomitant care

All patients are allowed to receive concomitant care as deemed necessary by their treating physician. In case concomitant care requires the removal of the cast during the first 3 weeks after tenotomy, and no new cast can be applied within 24 h of removal, the patient will be removed from the trial.

### Intervention allocation

The Indus Hospital Research Center (IHRC) will prepare 244 randomization envelopes according to the SNOSE-protocol [25] at their office before the start of enrollment of patients in the clinic. Only the exact number of envelopes necessary to carry out this study will be prepared. The envelopes are sequentially numbered and contain group allocation information for the patient allocation. Group information will be inserted in the envelopes using permutated blocks of random sizes according to the SNOSE-protocol (sequentially numbered, opaque sealed envelopes) [25]. The application of the SNOSE-protocol makes it impossible to predict which group allocation will be in the upcoming envelope when allocating envelopes according to their sequential numbering and thus protects concealment of the group allocation. However, this technique also assures a 1:1 randomization of the participants at the end of the study.

All patients who meet the inclusion criteria and have an informed consent form signed by a parent will be randomized. Randomization of patients and group allocation will happen in the clinic before the tenotomy takes place. No stratification of patients takes place during the group allocation process. Each envelope will contain a piece of aluminum foil, a piece of carbon paper, and a paper containing group information.

The study team member takes the envelop with the next available sequence number when a new patient registers for the study. Before opening the envelope, a study team member will write the MR number, name, date, and study ID of the patient on the envelope. The carbon paper inside the envelope will transfer the patient details onto the paper inside the envelope, avoiding any possibility for fraud or accidental group misplacement.

In case of retraction of informed consent for the study or surgical procedure, the envelope will be returned to IHRC. When all available envelopes have been used, IHRC will provide a new set of randomization envelopes, equal to the number of returned envelopes. This procedure assures that 1:1 randomization is maintained throughout the study.

### Blinding

#### Blinding/masking

Assessment of primary and secondary outcomes will be assessed by clinical officer on duty at the Pehla Qadam clubfoot clinic and will be blinded to group allocation. The medical officer sees all children attending the clinic and will not be aware if the child is enrolled in the trial and in which group his/her foot/feet are randomized and if the child received an Achilles tendon tenotomy or not. The program coordinator will extract the necessary data for the trial from the Pehla Qadam logbooks after every clinic day as to protect the blinding at the assessor level during clinic.

Given the nature of the intervention, the physician doing the tenotomy cannot be blinded to group allocation. Parents/caregivers can be informed about group allocation if they wish to know, at the time the tenotomy is performed.

#### Emergency unblinding

Emergency unblinding is not applicable in this trial. The medical officer assessing the patient’s foot/feet at 3 weeks and 3 months post-tenotomy is the only provider blinded to group allocation in this trial. If he/she believes the child requires additional assessment, because of inadequate progression during treatment, he/she can refer the child to the treating orthopedic surgeon for further assessment. The treating orthopedic surgeon is aware of the group allocation and will do all necessary assessments and will put the child’s health before the trial. This may include unenrolling the child from the trial, if necessary. The medical officer on duty will remain blinded to the group allocation for this respective child.

### Data collection

#### Data collection methods and participant retention

The primary and secondary outcome data collected for this trial is data that is already being collected as part of the standard follow-up of clubfoot patients at the Pehla Qadam clubfoot clinic. Therefore, no additional interventions will be done to increase the retention of participants beyond the counseling already offered to parents/caregivers of a child with clubfoot.

Assessors will not require any additional training beyond the training they receive to assess children in the clinic. The program coordinator will extract the necessary data for the trial from the Pehla Qadam logbooks after each clinic day and record it in the data tracking sheet.

### Data analysis and statistical methods

#### Interim analysis

During the RCT, one safety check will be done when 50% of the required feet are enrolled and the 3-month follow-up has been completed for these feet. The data sheet will be shared by the program coordinator with the Indus Hospital Research Center (IHRC) who will perform the interim analysis. The IHRC is not involved in the direct implementation of the trial nor in performing tenotomies or supervising residents in the clinical setting. The results of the interim statistical analysis will not be shared with the research team, if no statistical difference (non-inferiority) is observed between both groups or if the blade tenotomy shows to be statistically significantly inferior to the needle tenotomy. The trial will continue as planned in both of those cases. In case that a statistically significant difference was to be observed, and the needle tenotomy is inferior to the blade tenotomy, the trial will be ended based on the assumption that non-inferiority cannot be proven and patients should not be needlessly exposed to an inferior tenotomy technique.

#### Outcomes

Group A will be compared to group B for the primary and secondary analyses. A multi-level mixed-effects regression model will be used to assess the outcomes. An overview of the different variables to be assessed can be found in Table 1. A linear regression model will be used for the continuous variables and a logistic regression model for the binary variables. If a child received a tenotomy on both feet, these two feet will be clustered, and the analysis must make an adjustment for the lack of independence between measures on these two feet. A mixed-effects model allows for the inclusion of a random subject effect to the regression model, accounting for correlation between pairs of feet on the same child and also assessing the treatment effect from both between-subject differences and within-subject differences. Stata Version 17 (StataCorp, TX, USA) will be used for the analysis. A *p*-value < 0.05 will be considered significant. We will use the one-tailed *p*-value for all our analyses.

**Table 1:** Clinical outcomes and proposed statistical analysis

Variable/outcome	Hypothesis	Outcome measure	Method of analysis
**Primary outcomes**			
Dorsiflexion at 3 weeks post-tenotomy	Equal range of dorsiflexion	Degrees of dorsiflexion with 5° increments	Multi-level mixed effects linear regression
Dorsiflexion at 3 months post-tenotomy	Equal range of dorsiflexion	Degrees of dorsiflexion with 5° increments	Multi-level mixed effects linear regression
**Secondary outcomes**			
Pirani score at 3 weeks post-tenotomy	Equal score	Pirani score with 0.5-point increments	Multi-level mixed effects linear regression
Pirani score at 3 months post-tenotomy	Equal score	Pirani score with 0.5-point increments	Multi-level mixed effects linear regression
Minor complications at 3 weeks post-tenotomy	Equal count	Number of minor complications	Multi-level mixed effects logistic regression
Minor complications at 3 months post-tenotomy	Equal count	Number of minor complications	Multi-level mixed effects logistic regression
Major complications at 3 weeks post-tenotomy	Equal count	Number of major complications	Multi-level mixed effects logistic regression
Major complications at 3 months post-tenotomy	Equal count	Number of major complications	Multi-level mixed effects logistic regression
*Complication subgroup analysis*	*Equal count*	*Number of specific type of complications*	Multi-level mixed effects logistic regression

Non-inferiority will be established if the confidence interval for the needle group does not cross the non-inferiority margin. In practice, this means a confidence interval including only values < 4. If the confidence interval includes the value 4, the trial will be determined inconclusive. If the confidence interval only values > 4, the needle tenotomy technique will be considered inferior to the blade technique.

#### Analysis population and missing data

We will test for non-inferiority using the “intention-to-treat” approach. All participants will be assessed as part of the group they were randomized to. Children who were randomized to group A: needle tenotomy and received a blade tenotomy instead because of a decision made at the treating physician’s discretion are excluded from the trial. Cross-overs from the blade to the needle tenotomy group are not a possibility in our trial, given that the blade tenotomy is the gold standard and the “experimental” needle tenotomy is not performed based on parents’ request or physician’s preference. Therefore, there is no valid reason to assess the data in a “per-protocol” fashion.

As stated earlier, given the follow-up duration of 1 year, we expect a drop-out rate of about 10%, and the sample size will be adjusted for this expected loss-to-follow-up. Previous research shows that drop-out is predominantly related to a mix of socio-economic and contextual factors and much less related to the level of correction of the deformity or the satisfaction of the parents [18, 23]. Missing data is therefore considered to be missing at random (MAR) and no dummy variable creation or imputation will be done as the mixed effects regression models provide valid estimates under MAR.

Children who missed out on both follow-up consultations and for whom we were not able to schedule a follow-up consultation within 6 months of the tenotomy will be excluded from the trial.

### Protocol amendments

Any modifications that may impact the patient or the core structure of the study will require a formal amendment of the protocol and a formal approval of the IRB. Certain administrative changes to the protocol will require a formal approval by the IRB as well, as per the internal regulations at the Indus Hospital and Health Network.

The original protocol as submitted in September 2019 can be found in Additional file 2. The initial protocol already included two sample sizes for this study: one sample size for the pilot study and a sample size for the full RCT. After completion of the pilot study, the PI was changed to Dr. Mansoor Ali Khan, which required formal approval by the IRB as per local regulations, and the IRB was informed in writing of the continuation of the study as a full RCT with the full sample size as described in the original protocol. In October 2022, the final version of the protocol was created with a consolidated and agreed upon statistical analysis plan. This amendment did not require formal approval from the IRB.

### Recruitment

Recruitment will take place at the Pehla Qadam clubfoot clinic in Karachi, Pakistan. When a patient reaches the stage in his/her treatment where an Achilles tendon tenotomy is indicated, the treating physician will inform the program coordinator. The program coordinator will explain the trial to the parents/caregiver and initiate the process of taking consent as explained earlier. Recruitment will be ongoing until the full sample size of either the pilot study or the full RCT is reached using a non-probability consecutive sampling technique.

### Data management, access and confidentiality

All data collected as part of the standard treatment protocol at the Pehla Qadam clubfoot clinic is initially collected on paper and then subsequently entered in an electronic record by the program’s data management team. For the purposes of this trial, the program coordinator will allocate each enrolled foot a specific tracking number that will only be used for this trial. Only the program coordinator has access to the file linking the trial tracking number to a patient’s individual medical record number. A separate data collection sheet will be developed where the data for each enrolled foot will be collected based on the data entered into the electronic records of the Pehla Qadam clubfoot clinic. Since no additional data is collected as part of this trial, beyond the data that is recorded as a standard follow-up practice, no additional data management measures are necessary.

All data entered in the data collection sheet for this trial will be checked for errors by a program data management team member, other than the program coordinator, to assure data quality. All data will be backed-up electronically following the same scheduled data back-ups of all other medical data at the Indus Hospital and Health Network. No off-site data storage will be allowed.

The program coordinator will update the research team at least every 3 months about the number of feet enrolled in the trial and the expected time required until completion of enrollment.

The signed informed consent forms and envelopes used for the allocation procedures are stored in a locked cabinet at the Indus Hospital Research Center (IHRC) archives according to the safety procedures of the Indus Hospital and Health Network. The data will be stored for a minimum of 7 years after completion of the study, for both the pilot as well as the full RCT study. If a participant or his parent/caregiver decides to retract their informed consent for participation in the trial, only the initially signed informed consent form and a note logging the retraction will be kept. All other data relating to this participant will be destroyed and will no longer be part of the trial records.

Only de-identified data, using the trial tracking number for each foot instead of a personal identifier like the medical record number, will be shared with the research team. Age at time of tenotomy and at time of the two follow-up visits will be calculated by the program coordinator as to avoid sharing identifiable data such as a date of birth or a date of visit.

If a participant missed one of the required follow-up visits his/her parent/caregiver will be contacted to remind them of the importance of follow-up during clubfoot treatment. They will receive the standard phone call any patient in the clubfoot clinics receives when they miss an appointment. If a patient has not returned for follow-up after within 6 months of his/her last scheduled follow-up appointment, the patient’s enrolled foot/feet will be labeled as “lost to follow-up” for both the program’s purpose as well as for the trial’s purposes.

Participants’ data, including de-identified data, will not be shared with anyone outside of this trial without formal written consent of the parents/caregiver or the participant him/herself once they reach the age of 18 years.

### Harms and adverse events

Data on adverse events will be collected from the moment the patient enrolls in the trial. In case an adverse event takes place before the tenotomy has taken place, the adverse event will be recorded as “unrelated to trial.” If an adverse event takes places after the tenotomy has taken place, it will be recorded and examined for potential causality to the trial intervention. An adverse event that meets the criteria of a “serious adverse event” will be reported to the IRB for further evaluation. A serious adverse event is defined as a life-threatening condition, a condition inflicting severe or permanent disability or needing an additional surgical intervention beyond the standard treatment plan. Serious adverse events happening after the 12-month follow-up meeting will not be reported to the IRB unless the treating physician or the program coordinator suspects a causal relationship between the trial and the adverse event.

The study team will monitor for the following tenotomy-related adverse events: excessive bleeding, neurovascular damage, infection requiring antibiotic treatment, necessity to reperform the tenotomy after 3 weeks because of incomplete sectioning of the Achilles tendon.

### Auditing and data monitoring

Auditing will be done by the Indus Hospital Research Center (IHRC) as part of their oversight role for all research studies and trials that are ongoing at the Indus Hospital and Health Network. They will review informed consent forms, whether children meet the enrollment criteria, and if the data collection and management meet the required quality and data protection standards. None of the members of the IHRC are directly involved in the implementation or analysis of the data for this trial beyond the interim analysis. There will be no independent non-hospital affiliated audit because of the absence of such a structure in Pakistan.

The local context in Karachi, Pakistan, did not require the establishment of a data monitoring committee (DMC) for a low-risk trial. We therefore did not establish a DMC.

### Ancillary and post-trial care

The data collected for the purposes of this trial cannot be reused for other studies or purposes without the formal written consent of the parents/caregiver or the patient him/herself once he/she reaches the age of 18 years. No tiered informed consent form will be used. The data collected as part of this trial is also collected as part of the wider Pehla Qadam clubfoot clinic data collection effort. Reuse of the data collected as part of the Pehla Qadam clubfoot clinic data collection efforts is at the discretion of the Pehla Qadam clubfoot clinic and Indus Hospital and Health Network’s discretion and falls beyond the scope of this protocol.

All children will continue their standard care free-of-cost at the Pehla Qadam clubfoot clinic after their participation in the trial if they wish so. In case any harm done to one of the participants, the required medical care will be offered free-of-cost at the Indus Hospital and Health Network to the participant.

### Dissemination policy

#### Trial results

All abstracts, posters, dissertations, journal manuscripts, or other publications based on the data collected during this trial will be shared with the entire research team for approval before submission or presentation. The submission should clearly state whether the data is from the pilot study or from the full RCT in the title. The research team does supports individual endeavors to present this research at various national and international fora in the format of a poster or oral presentation. The research team plans to publish the results of the full RCT in an international journal and on the ClinicalTrials.gov website once the trial has been completed.

No data from the interim safety check will be shared in any form with anyone outside of the designated people earlier in this protocol. Data from both the pilot study and the full RCT can only be shared outside the research team, once enrollment, follow-up, and data analysis are complete.

Data will be shared with participants and/or their parents/caretakers as long as they are minors upon request. No public debriefing or results sharing session for participants and their relatives will be done.

#### Authorship

Authorship on publication of the results of the full RCT in an international journal will be offered to everyone who contributed significantly to the design, implementation, conduct, or evaluation of results of the trial. All authors on the final manuscript should be able and willing to take full responsibility for the trial conduct and its results and should be able to explain and interpret the results. All authors need to fulfil the ICJME authorship criteria. Both JGS and MP need to acknowledge the roles and contributions of the entire research team in their respective dissertations in the proper format proposed by their institutions.

Authorship on abstract submissions, poster presentations, and podium presentations is allowed to diverge from the authorship and authorship order on this protocol as well as the final manuscript. Authorship for this type of submissions should reflect the input given and contributions made to this respective submission, while also respecting the overall contributions made by the team to entire trial. No author can be added to any submission or publication without their formal approval and agreement.

#### Reproducible research

Commitments are made by the research team to support efforts towards reproducible research, by publishing this protocol open access. All reasonable requests for full data access, access to the statistical analysis code, and the full study report, after completion of the trial, will be considered by the research team.

## Discussion

This study provides critical evidence in the assessment of the efficacy of a 22-gauge needle tenotomy for Achilles tendon tenotomies in clubfoot. To our knowledge, it will be the first scientifically rigorous randomized controlled trial with a sufficiently large sample size to assess the non-inferiority of the needle technique. Additionally, as this trial is set in a middle-income country, it can serve as a technical example for other middle-income country researchers aspiring to set up clinical trials in their settings, as a source of education for high-income researchers showcasing that high-quality scientifically rigorous research is being produced in low- and middle-income countries and as a source of aspiration for the pediatric orthopedic community that high-quality trials can help inform our treatment plans and move our field forward in an evidence-based manner.

Several design aspects merit discussion in this section. We chose the control-group intervention to be blade tenotomies performed under local anesthesia in an outpatient setting. This decision was made so that the only aspect of the treatment being randomized is the instrument with which the tenotomy is performed, i.e., the blade or the needle. We acknowledge that by not taking the setting (operating theater or outpatient clinic) and the type of anesthesia (local or general) into randomization in our trial, we minimize the impact of potential confounding factors in our study but also exclude certain factors from randomization that may potentially have an indirect impact on the clinical outcome measures recorded in this trial.

In the Pehla Qadam clinic, 3-month follow-up is the standard follow-up offered to patients after an Achilles tendon tenotomy. This 3 months’ timeframe is based on the theory that the tenotomy has healed after 3 months and that the dorsiflexion range after the tenotomy will no longer change at this point [26]. Therefore, we considered this a justifiable length of follow-up to make an informed claim as to the clinical effects and non-inferiority of the needle tenotomy. However, since many children receive their tenotomy before the age of 1 year, many children are still non-walking at the 3 month follow-up mark. It is unknown to what extent walking influences the dorsiflexion range after an Achilles tendon tenotomy in a child with treated clubfoot. It therefore remains to be kept in mind that walking has a potential influence of unknown magnitude on the range of dorsiflexion after a needle tenotomy that is not evaluated in this trial.

We hypothesize that the needle tenotomy technique will be non-inferior to the gold standard blade tenotomy technique, meaning that the mean difference in range of dorsiflexion is < 4°. By showing the non-inferiority of the technique, we provide the evidence needed for clinics around the globe to roll out the technique in their clinical settings if they would like to do so. Specifically for low-resource settings, we believe that this trial can provide important evidence to shift their practice, since we know that needle tenotomy is cheaper, more accepted by the parents, and easier to perform [15].

## Supplementary files

### Model consent form

The English version of consent form used in this study can be found in Additional file 1 of this protocol.

### Initially approved protocol

The initial version of the protocol approved by the IRB at the start of this study can be found in Additional file 2 of this protocol.

## Trial summary

### Trial registration summary

A summary of the trial and its registration information can be found in Table 2.

**Table 2:** Trial summary

Data category	Information
Primary registry and trial identifying number	ClinicalTrials.gov NCT04897100
Date of registration in primary registry	May 21, 2021
Secondary identifying numbers	N/A
Source(s) of monetary or material support	The Indus Hospital & Health Network, Karachi, Pakistan
Primary sponsor	The Indus Hospital & Health Network, Karachi, Pakistan
Secondary sponsor	N/A
Contact for public queries	Sadia Ahmed, sadia.ahmed@tih.org.pk
Contact for scientific queries	Dr. Mansoor Ali Khan, The Indus Hospital & Health Network, Karachi, Pakistan mansoor.khan@tih.org.pk Dr. Manon Pigeolet, Université Libre de Bruxelles, Brussels, Belgium, manon.pigeolet@ulb.be
Public title	Outcome and Complications After Percutaneous Needle Versus Blade Achilles Tenotomy in Clubfoot Treated With the Ponseti Method
Scientific title	A single-center, single-blinded, randomized, parallel-group, non-inferiority trial to compare the efficacy of a 22-gauge needle versus a 15 blade to perform an Achilles tendon tenotomy in 244 clubfeet – study protocol
Countries of recruitment	Pakistan
Health condition(s) or problem(s) studied	Clubfoot; Achilles tendon tenotomy
Intervention(s)	Treatment group A: Achilles tendon tenotomy with 22-gauge needle Treatment group B: Achilles tendon tenotomy with 15 blade
Key inclusion and exclusion criteria	Ages eligible for study: < 36 months Sexes eligible for study: both Accepts healthy volunteers: no Inclusion criteria: Idiopathic clubfoot requiring Achilles tendon tenotomy after a full casting cycle Exclusion criteria: syndromic clubfoot, prior treatment for clubfoot
Study type	Interventional Allocation: block randomization with a 1:1 allocation Intervention model: parallel assignment Masking: Single-blinded study. Data collectors during follow-up are unaware of which intervention the patient received Primary purpose: improve treatment Phase IV
Date of first enrolment	21 May 2021
Target sample size	244 feet
Recruitment status	Recruiting
Primary outcome(s)	Degrees of dorsiflexion at 3 weeks and 3 months post-tenotomy
Key secondary outcomes	Minor and major complications at time of tenotomy, 3 weeks and 3 months post-tenotomy

### Protocol version

Version date: March 2023.

The protocol revision chronology is presented in Table 3.

**Table 3:** Protocol revision chronology

September 2019	Original
March 2023	Amendment of statistical analysis plan and final determination of sample size using a non-inferiority margin

## Trial status

Enrollment in this trial began on 21 May 2021 and is estimated to be finished by September 2023. Follow-up of patients and data collection is planned to be finished by the end of December 2023. This protocol version is version 2, dated 31 March 2023. Enrollment of participants is ongoing at the time of submission.

### Supplementary Information

**Additional file 1.** English version of consent form used in this study.

**Additional file 2.** Initial version of the protocol approved by the IRB at the start of this study.

**Additional file 3.**

## Data Availability

The full dataset generated by this trial will be made available in de-identified form as a supplement to the publication of the RCT.
