# Supplementary material for: A single-center, single-blinded, randomized, parallel-group, non-inferiority trial to compare the efficacy of a 22-gauge needle versus a 15 blade to perform an Achilles tendon tenotomy in 244 clubfeet—study protocol
Source: Trials. 2023 Oct 31;24:701. doi: 10.1186/s13063-023-07728-9 (PMC10617068; doi:10.1186/s13063-023-07728-9)
Supplement: Supplementary file 1 — Additional file 1. English version of consent form used in this study. [file 13063_2023_7728_MOESM1_ESM.docx]

Supplement 1

**CONSENT FORM**

**Study Title: Outcome and complications after percutaneous needle versus blade Achilles tenotomy in clubfoot treated with the Ponseti method**

**What you should know about the study:**

- You are being asked to join a research study
- This consent form explains the research study and your part in it
- You are a volunteer. You have a right to choose whether to participate or not in this study. If you decide to participate and later change your mind, you may do so without any penalty or consequences to your treatment here.

**Purpose of research project:**

Achilles tendon tenotomy (a minor cut) is an important part of the Ponseti method. Most babies need this cut before the final cast is put on. It usually be performed in an out-patient setting under local anesthesia using a needle or a blade**.** The doctor releases the tendon with a minor cut from the baby’s heel. The cut is so small that it does not need stitches and heals while the child wears the 3 weeks final cast. Through this study, we aim to compare the clinical outcomes (mobility of the foot) and complication rate in children receiving either a blade or a needle percutaneous tenotomy. We will be requesting 244 people undergoing tenotomy to be part of our study

**What you will be asked to do in this study**

If you agree to be part of this study, your child will be placed in one of two groups randomly.

Group A: If your child is placed in this group, the cut will be performed with a needle.

Group B: If your child is placed in this group, the cut will be performed with a blade.

After the cut, a doctor will apply the final cast for about 3 weeks to the children of both groups. You will be part of this study until 6 months after the removal of the final cast. 6 months after the removal of the final cast, the doctor will do routine check-up which are part of the treatment of your child and will collect some final measurements about the mobility of your child’s foot for this study.

**Privacy:** We assure you that your research information will be kept secret. We will use ID numbers instead of your child’s name. Your child’s name will not be given to anyone without your consent. Study doctor, researchers from Indus Hospital will be able to look at your medical records. It is possible that research papers may be published for scientific purposes; however, your child’s name nor that of any other participant will not be used.

**Risks and Discomfort:** Regardless of the group your child is involved in, there is no risk involved because of this research.

**Benefits:** knowing the clinical outcomes and complication rates of both of the techniques would benefit children with clubfoot in the future.

**Alternatives:** You have the choice of not being part of the study

**What happen if you choose not to be part of the study?**

If you decide not to participate in this research, your child’s treatment at the hospital will continue as usual. Even after signing this consent form, you may stop participating at any time.

**Cost of taking part in research:** You do not have to pay for taking part in this study.

**Payment for taking part in research**: You will not get money to be part of this study.

**Who do I call if have questions or problem?**

If you have questions about this research, you can contact Dr. Mansoor Ali Khan He can be contacted at the The Indus Hospital, (Mobile 0300 827 2693)

If you have questions about your rights in a research study as a volunteer, call or contact the Interactive Research & Development-IRB office between office hours on Monday to Friday 9am – 5pm at the Indus Hospital (Mobile 0300 827 2693)

**SIGNATURES**

If you agree to participate in this study, please sign this form. You will receive a copy of this form.

Sign of study volunteer/participant: _____________________________ Date:___________________

Sign of person taking consent: _____________________________ Date: __________________
