# Supplementary material for: A single-center, single-blinded, randomized, parallel-group, non-inferiority trial to compare the efficacy of a 22-gauge needle versus a 15 blade to perform an Achilles tendon tenotomy in 244 clubfeet—study protocol
Source: Trials. 2023 Oct 31;24:701. doi: 10.1186/s13063-023-07728-9 (PMC10617068; doi:10.1186/s13063-023-07728-9)
Supplement: Supplementary file 2 — Additional file 2. Initial version of the protocol approved by the IRB at the start of this study. [file 13063_2023_7728_MOESM2_ESM.docx]

# Supplement 2

**Outcome and complications after percutaneous needle versus blade Achilles tenotomy in clubfoot treated with the Ponseti method**

Synopsis

INTRODUCTION

Achilles tendon tenotomy is an integral part of the Ponseti method, aimed at correcting residual equinus after correction of the adductus deformity([1](#_ENREF_1)). Tenotomy rates ranging from 70-80% after full cycle of castings have been reported in literature([1](#_ENREF_1), [2](#_ENREF_2)). Percutaneous tenotomy is the gold standard, which can usually be performed in an out-patient setting under local anesthesia using a scalpel blade([3](#_ENREF_3)). A complication rate of 2% (mainly neurovascular injury)has been reported in literature, with accidental sectioning of the peroneal artery being the most common([4](#_ENREF_4)). Development of a pseudo-aneurysm after accidental sectioning of the peroneal artery has been reported in a case report; this delayed further clubfoot treatment([5](#_ENREF_5)).

Percutaneous needle tenotomy has been described by some authors as an alternative technique with very favorable results in a population before walking age([6](#_ENREF_6), [7](#_ENREF_7)).This technique use a large-gauge (16-19 G) needle to percutaneously cut the Achilles tendon([6-8](#_ENREF_6)). Although bleeding has been reported following this technique with similar rates as for the percutaneous blade technique, no major complications have been reported as yet using the needle technique([6](#_ENREF_6), [7](#_ENREF_7)).

A high correction rate of residual equinus has been reported in literature using percutaneous Achilles tendon tenotomy ([9-11](#_ENREF_9)). A limited amount of patients present with a dorsiflexion of less than 15° after 1 year follow-up, however this does not affect the gait or the functionality of the foot([10](#_ENREF_10)). None of these studies however specify the technique used for the percutaneous tenotomy. Considering the very limited reported use of percutaneous needle tenotomy, it can be presumed that the regular blade tenotomy technique has been used.

Percutaneous needle tenotomy has been described as a safe intervention in other clinical settings as well, including percutaneous needle tenotomy of the quadriceps tendon in congentinal knee dislocation in newborns ([12](#_ENREF_12)) and for superficial tendon tenotmies, including Achilles tendon tenotomy, in stroke patients with tendon contractures([13](#_ENREF_13)). No contraindications have been reported for this technique to date([12](#_ENREF_12), [13](#_ENREF_13)).

To date, 1 randomized control trial comparing percutaneous needle versus blade tenotomy has been published by a tertiary hospital in India in children undergoing Achilles tendon tenotomy under the age of 1 year([14](#_ENREF_14)). Complication rate and post-operative Pirani scores were recorded for all patients. In this study fifty five club feet were undergone the treatment procedure and followed for one year. Out of fifty five feet twenty seven were treated with blade tenotomy and twenty eight feet were treated with needle tenotomy. Pre-treatment Pirani score was 5.62 vs 5.58 in blade and needle tenotomy group respectively. One patient in needle tenotomy group developed relapse due to poor application of Denis Browne splint. Post treatment mean Pirani score in blade and needle tenotomy group was 0.26 and 0.31 respectively. Author did not find any significant statistical difference between the two groups of percutaneous tenotomy (p>0.05). Although no information about dorsiflexion rate is mentioned. No information is given about demographic features of both groups and possible significant differences between them or about the randomization strategy. No information is given about the initial number of patients enrolled in the study and the drop-out rate. However, this study can be considered as a pilot study for our study, considering the similar setting and study population. Through our study, we aim to compare the clinical outcomes and complication rate in children receiving either a blade or a needle percutaneous tenotomy.

Rationale:

The ponseti method has become the main accepted treatment for clubfoot globally. It involves achilles tendon tenotomy which is usually done by blade in majority of settings. In low resource settings a needle tenotomy may be more accesible, cheaper and easier to provide by health care provider as compared to blade tenotomy. However using blade can cause many complications such as larger wound, possible scar mark, excessive bleeding and pain. Moreover fear of the parents regarding the blade tenotomy procedure can be very high, which causes great anxiety among them. We hope that proving needle tenotomy equal to gold standard blade tenotomy, can be alternative to overcome these complications, more acceptable to parents and may creat better access to clubfoot care in low-resourse settings.

Objectives:

Through this study, we aim to compare the clinical outcomes and complication rate in children receiving either a blade or a needle percutaneous tenotomy.

Hypothesis:

Percutaneous needle tenotomy has better clinical outcomes and less complication rate as compared to Percutaneous blade tenotomy

Operational Definations:

# Clubfoot (congenital talipes Equinovarus): Defined as fixed position of one or both feet in adduction, supination and varus that is inclined inward, axially rotated outwards and pointing downwards ([15](#_ENREF_15)).

**Ponseti method:** The Ponseti method is a conservative treatment of clubfeet babies with proper manipulative techniqeus of foot followed by plaster cast application to achieve best and safest correction of clubfoot deformity([16](#_ENREF_16)).

**Tenotomy:** A tenotomy is a surgical act which involves the division of a tendon.

**Pirani Scoring**: It is the subjective criterion which gives the numerical value to assess the severity of clubfoot deformity. It value ranges from 0-6.([17](#_ENREF_17))

Materials and Methods:

Study design: Randomized Control Trial (RCT).

Study arms:

1. Group ‘A’ – Percutaneous needle tenotomy
2. Group ‘B’ – Percutaneous blade tenotomy.

Setting: The study will be conducted at The Indus Hospital Korangi campus, Karachi.

Duration of study: Six months after CPSP and IRB approval.

Sample size:

Sample size was calculated using WHO sample size determination software with the following assumptions

Alpha=5%

Power 80%

Success rate of blade tenotomy=100%([14](#_ENREF_14)).

Success rate of needle tenotomy=96.7%([14](#_ENREF_14)).

Required sample size=312 (156 in each arm).

_____________________________________________________________

Alpha=5%

Power 80%

Prevalence of Complications due to blade tenotomy= 11.11%([14](#_ENREF_14)).

Prevalence of Complications due to needle tenotomy= 3.6%([14](#_ENREF_14)).

Required sample size= 252 (126 in each arm)

On average we do 60 tenotomies per year hence the sample is large unachievable. We will enroll a total of 60 patients in our study (30 in each arm).

Sampling Technique: Non probability consecutive sampling.

Selection Criteria:

Inclusion criteria:

- Idiopathic clubfoot.
- Age less than or equal to 36 months at the time of tenotomy.
- Enrolled at the Pehla Qadam clinic at The Indus Hospital in Karachi.
- Fully corrected Adductus deformity with residual equinus after a full casting cycle.
- Completing routine follow up for 3 months post tenotomy.

Exclusion criteria:

- Refusal of parents to enroll child into this study.
- Syndromic clubfoot.
- Previous treatment for clubfoot (surgical or non-surgical) received.
- Underlying medical conditions unrelated to clubfoot that may serve as a contra-indication, this decision will be left on the discretion of the treating orthopedic surgeon.

Data Collection Procedure:

Study will be commenced after approval from CPSP and IRB. Children will be assessed for eligibility in Pehla Qadam clinic. Written informed consent will be taken from the parents of all the eligible children. All the children will then be randomly divided into one of the two study arms. Randomization will be done through SNOSE protocol ([18](#_ENREF_18)). Indus Hospital Research Center will prepare SNOSE envelopes that are sequentially numbered and opaque. Envelopes contains aluminium foil, carbon paper and a paper containing group information. Before opening these envelopes a study team member will write all the details like MR number, name, date, and study ID on the top of the envelope. The carbon paper inside the envelopes will then transfer all these details on the paper inside the envelope.

In group A tenotomy will be done through needle and in group B tenotomy will be done according to the standard protocol that is using blade. Foreach children undergoing tenotomy, pre and post tenotomy Pirani’s severity scoring will be recorded.

**Clinical endpoints and follow-up**

Patients with residual equinus and corrected adductus deformity will undergo one of the 2 proposed methods for Achilles tendon tenotomy. A measurement tool (jig) set at 20 degrees dorsiflexion will be needed. If a child’s foot can be easily positioned in the jig at the decided time points, then this will demonstrate correction of equinus. Time points for measurement will be at time of index procedure, 3 weeks after the procedure when the final cast is taken off and at the regular 3-month follow-up consultation. Complications like neurovascular damage in the heel region, skin and wound problems will be recorded for statistical purposes.

Statistical Analysis:

Data will be entered using SPSS version 25.0 Mean ± SD/Median (IQR) will be computed for all the quantitative variables like age, pirani score, and dorsiflexion. Frequency and percentage will be computed for all the qualitative variables like gender, idiopathic clubfoot, foot affected, and complications. Independent sample t-test/Mann-Whitney U test will be applied as appropriate to assess difference in age, pirani score, and dorsiflexion between both the study groups. Chi-square/Fisher-exact test will be applied as appropriate to assess association between gender and complications. Effect modifiers will be controlled through stratification of idiopathic clubfoot, and foot affected. P-value <0.05 will be considered statistically significant.

**References**

1. Scher DM, Feldman DS, van Bosse HJ, Sala DA, Lehman WB. Predicting the need for tenotomy in the Ponseti method for correction of clubfeet. J Pediatr Orthop. 2004;24(4):349-52.

2. De Mulder T, Prinsen S, Van Campenhout A. Treatment of non-idiopathic clubfeet with the Ponseti method: a systematic review. J Child Orthop. 2018;12(6):575-81.

3. Hedrick B, Gettys FK, Richards S, Muchow RD, Jo CH, Abbott MD. Percutaneous heel cord release for clubfoot: a retrospective, multicentre cost analysis. J Child Orthop. 2018;12(3):273-8.

4. Dobbs MB, Gordon JE, Walton T, Schoenecker PL. Bleeding complications following percutaneous tendoachilles tenotomy in the treatment of clubfoot deformity. J Pediatr Orthop. 2004;24(4):353-7.

5. Burghardt RD, Herzenberg JE, Ranade A. Pseudoaneurysm after Ponseti percutaneous Achilles tenotomy: a case report. J Pediatr Orthop. 2008;28(3):366-9.

6. Evans A, Chowdhury M, Rana S, Rahman S, Mahboob AH. ‘Fast cast’and ‘needle Tenotomy’protocols with the Ponseti method to improve clubfoot management in Bangladesh. J Foot Ankle Res. 2017;10(1):49.

7. Rahman MS, Alam MK, Shahiduzzaman M, Rahman A. Percutaneous needle tenotomy for Ponseti technique in the management of Congenital Talipes Equinovarus (CTEV). J Dhaka Med Coll. 2014;23(1):55-9.

8. Patwardhan S, Shyam A, Sancheti P. Percutaneous Needle Tenotomy for Tendo-achillis Release in Clubfoot–Technical Note. J Orthop Case Rep. 2012;2(1):35.

9. Alam MT, Akber EB, Alam QS, Reza MS, Mahboob AH, Salam SI, et al. Outcome of Percutaneous Tenotomy in the Management of Congenital Talipes Equino Varus by Ponseti Method. Mymensingh Med J. 2015;24(3):467-70.

10. Liu YB, Jiang SY, Zhao L, Yu Y, Tao XC, Zhao DH. Functional Assessment of the Foot Undergoing Percutaneous Achilles Tenotomy in Term of Gait Analysis. Biomed Res Int. 2016;2016:1973403.

11. Noh H, Park SS. Predictive factors for residual equinovarus deformity following Ponseti treatment and percutaneous Achilles tenotomy for idiopathic clubfoot: a retrospective review of 50 cases followed for median 2 years. Acta Orthop. 2013;84(2):213-7.

12. Patwardhan S, Shah K, Shyam A, Sancheti P. Assessment of clinical outcome of percutaneous needle quadriceps tenotomy in the treatment of congenital knee dislocation. Int Orthop. 2015;39(8):1587-92.

13. Coroian F, Jourdan C, Froger J, Anquetil C, Choquet O, Coulet B, et al. Percutaneous Needle Tenotomy for the Treatment of Muscle and Tendon Contractures in Adults With Brain Damage: Results and Complications. Arch Phys Med Rehabil. 2017;98(5):915-22.

14. Choubey R, Jain A. Comparison of percutaneous tenotomy techniques for correction of equinus deformity in Congenital Talipes Equino Varus (CTEV) in children: a randomized clinical trial. J Evol Med Dent Sci. 2015;4(57):9865-70.

15. Miedzybrodzka Z. Congenital talipes equinovarus (clubfoot): a disorder of the foot but not the hand. J Anat. 2003;202(1):37-42.

16. Ponseti IV. Common errors in the treatment of congenital clubfoot. Int Orthop. 1997;21(2):137-41.

17. Anshuman R, Singh M, Jain BK, Verma N, Arora R. Correlation of Pirani score and Foot bimalleolar angle in the treatment of idiopathic congenital talipes equino varus by Ponseti method in infants. Acta Orthop Belg. 2016;82(4):861-5.

18. Doig GS, Simpson F. Randomization and allocation concealment: a practical guide for researchers. J Crit Care. 2005;20(2):187-91; discussion 91-3.
